# *Epicoccum nigrum* P16, a Sugarcane Endophyte, Produces Antifungal Compounds and Induces Root Growth

**DOI:** 10.1371/journal.pone.0036826

**Published:** 2012-06-04

**Authors:** Léia Cecilia de Lima Fávaro, Fernanda Luiza de Souza Sebastianes, Welington Luiz Araújo

**Affiliations:** 1 Brazilian Agricultural Research Corporation, Embrapa Agroenergia, Brasília, Distrito Federal, Brazil; 2 Department of Genetics, “Luiz de Queiroz” College of Agriculture, University of São Paulo, Piracicaba, São Paulo, Brazil; 3 Department of Microbiology, Biomedical Sciences Institute, University of São Paulo, São Paulo, São Paulo, Brazil; Auburn University, United States of America

## Abstract

**Background:**

Sugarcane is one of the most important crops in Brazil, mainly because of its use in biofuel production. Recent studies have sought to determine the role of sugarcane endophytic microbial diversity in microorganism-plant interactions, and their biotechnological potential. *Epicoccum nigrum* is an important sugarcane endophytic fungus that has been associated with the biological control of phytopathogens, and the production of secondary metabolites. In spite of several studies carried out to define the better conditions to use *E. nigrum* in different crops, little is known about the establishment of an endophytic interaction, and its potential effects on plant physiology.

**Methodology/Principal Findings:**

We report an approach based on inoculation followed by re-isolation, molecular monitoring, microscopic analysis, plant growth responses to fungal colonization, and antimicrobial activity tests to study the basic aspects of the *E. nigrum* endophytic interaction with sugarcane, and the effects of colonization on plant physiology. The results indicate that *E. nigrum* was capable of increasing the root system biomass and producing compounds that inhibit the *in vitro* growth of sugarcane pathogens *Fusarium verticillioides*, *Colletotrichum falcatum*, *Ceratocystis paradoxa*, and *Xanthomomas albilineans*. In addition, *E. nigrum* preferentially colonizes the sugarcane surface and, occasionally, the endophytic environment.

**Conclusions/Significance:**

Our work demonstrates that *E. nigrum* has great potential for sugarcane crop application because it is capable of increasing the root system biomass and controlling pathogens. The study of the basic aspects of the interaction of *E. nigrum* with sugarcane demonstrated the facultative endophytism of *E. nigrum* and its preference for the phylloplane environment, which should be considered in future studies of biocontrol using this species. In addition, this work contributes to the knowledge of the interaction of this ubiquitous endophyte with the host plant, and also to a better use of microbial endophytes in agriculture.

## Introduction

Endophytic microorganisms, primarily bacteria and fungi, inhabit, for at least one period of their life cycle, the interior of the host plant without inducing disease symptoms or producing external structures [1]. The bioprotective effects of these organisms, such as growth promotion and tolerance to herbivory and abiotic stress, are well-known for some temperate climate plants (*Poaceae*) [2], [3]. The diversity of endophytic fungi is greater in tropical regions. Plants in these regions are considered to be true reservoirs of fungal diversity [1], [4], [5], but the endophyte-plant interaction under these conditions is not yet fully understood [6]. In fact, the plant interior is now recognized as a prolific environment for the discovery of fungi with new biological activities [7], [8], [9], especially biocontrol capabilities [10], [11], [12]. Although endophytes have potential use in agriculture, the incomplete understanding of the biology of the endophyte-plant interaction presents impedes their wider use [2], [13].

Microorganisms that naturally associate with sugarcane, especially atmospheric nitrogen fixing bacteria and plant growth-promoting bacteria, have contributed to more productive agriculture with decreased environmental impact [14], [15]. The investigation of sugarcane endophytic bacterial communities and their related soil microbial populations has resulted in a greater comprehension of the bacterial population dynamics in the tropical agricultural environment [16] and the potential for the biological control of sugarcane pathogens [17], [18]. However, there have been few studies on the fungal communities associated with *Saccharum officinarum* [19], [20]. Our group has previously assessed the endophytic fungal communities associated with sugarcane [21], [22], [23] and their biotechnological potential [24], [25], [26], [27]. Notably, the ascomycete *E. nigrum* has been frequently isolated as an endophyte of sugarcane plants [21], [22], [23], [24].

*E. nigrum* Link (syn. *E. purpurascens* Ehrenb. ex Schlecht.) is a widespread mitosporic ascomycete (Dothideomycetes) that colonizes different types of substrates and is associated with plant primary decomposition [28]. Similar to other ubiquitous fungi, *E.* *nigrum* can display an endophytic lifestyle [29] in a variety of plants that are not taxonomically related [21], [30], [31], [32], [33], which suggests the development of adaptations to overcome the different types of plant defenses. *E. nigrum* is especially known for its biocontrol activity against pathogens, such as *Sclerotinia sclerotiorum* in sunflower [34], *Pythium* in cotton [35], phytoplasma in apple trees [36] and *Monilinia* spp. in peaches and nectarines [37], [38], [39], [40]. In spite of its biotechnological potential, little is known about the interaction of *E. nigrum* with plants [36], [41], and there have been no studies of the endophytic interaction of *E.* *nigrum* with tropical plants. Some studies of the *E. nigrum* endophytic interaction have been performed; for example, primers for the variable ITS1 and ITS2 regions were developed to detect endophytic *E. nigrum* in grapevines with and without phytoplasma symptoms [32]. In another study, the inoculation of an endophytic *E. nigrum* strain from an apple tree in the model plant *Catharanthus roseus* triggered defense responses against “*Candidatus* Phytoplasma mali” and reduced symptom severity [36]. These findings illustrate the potential for the use of endophytic *E. nigrum* in different host plants and warrant a further investigation of the physiological and molecular aspects of the interaction.

The aim of this work was to gain insight into the interaction of this common sugarcane endophyte with the host plant and to assess the antagonistic capacity of *E. nigrum* against *S. officinarum* plant pathogens. The growth responses of the sugarcane plants to colonization by *E. nigrum* were also investigated, and aspects of the lifestyle of this fungus are discussed.

## Results and Discussion

### *E. nigrum* Establishes a Facultative Endophytic Interaction, Preferentially Colonizes the Phylloplane, and Causes Transient Changes in the Sugarcane-associated Fungal Community

The basic aspects of the interaction of the common sugarcane endophyte *E. nigrum* with the host plant were investigated by inoculation on the leaves and roots of plants grown in a greenhouse and later re-isolation in a time-course experiment. In addition to monitoring *E. nigrum* colonization by isolation from disinfected and non-disinfected sugarcane organs over time, this approach permitted the comparison of the *E. nigrum* isolation frequency with the total isolation frequency of plant-associated fungi. Importantly, the Random Amplified Polymorphic DNA (RAPD) profiles of all of the re-isolated *E. nigrum* were the same as those of the original P16 strain (Figure S1).

**Figure 1:**
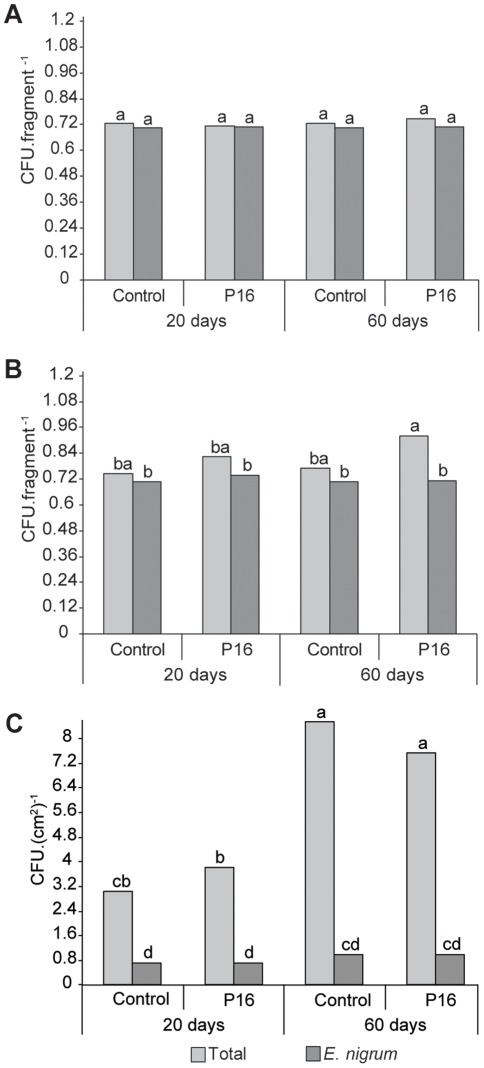
*E. nigrum* and sugarcane-associated fungi re-isolation from the phylloplane of sugarcane grown in greenhouse. *E.* *nigrum* and sugarcane-associated fungi were re-isolated 20 and 60 days after inoculation of the P16 endophytic strain on leaves of sugarcane plants. The *E. nigrum* isolation frequency was compared with the total isolation frequency of sugarcane-associated fungi. Isolation frequency of the endophytic fungi from leaves (a) and sheaths (b) is shown in CFU per leaf/sheath fragment. Isolation frequency of epiphytic fungi (c) is shown in CFU per cm^2^ and includes abaxial and adaxial surfaces of the leaf fragments. All the data were transformed with √ x + 0.5 and submitted to analysis of variance followed by Tukey’s test. Means followed by the same letter indicate that they were not statistically different (Tukey’s test, *P>5%*). Control indicates the non-inoculated plants, while P16 indicates plants inoculated with the *E. nigrum* P16 strain.

The isolation analysis of the disinfected sugarcane leaves and sheaths did not reveal significant differences in the number of *E.* *nigrum* CFUs recovered from these organs during the two sampling periods, as observed from the analysis of variance (Figure 1a and Figure 1b). *E. nigrum* was not isolated as an endophyte from the leaves and sheaths of the control plants in any sample period. In studying the effect of the inoculation with *E. nigrum* mycelia fragments on the endophytic fungal isolation frequency in these organs, the analysis also did not reveal differences in the number of CFUs recovered from the leaves over time (Figure 1a), but the number of CFUs recovered from the sheaths increased as the plant aged (Figure 1b). Most of the colonies obtained from the inoculated plants after disinfection were *E. nigrum*, which demonstrates the capacity of this fungus to endophytically colonize not only senescent leaves but also newly opened leaves on the sugarcane plants (not shown). These results also confirm previous reports on the sugarcane endophytic behavior of *E. nigrum* [21], [22] and indicate that *E. nigrum* is capable of disseminating to other tissues after the inoculation on leaves with mycelial fragments.

A different scenario was observed for the epiphytic environment. The number of *E. nigrum* CFU recovered from the leaf surface increased as the plant aged, as observed from the analysis of variance (Figure 1c). *E. nigrum* was recovered from the leaf surface of the control plants 60 days after inoculation (Figure 1c), which suggests that the inoculated plants served as a potential inoculum source in the greenhouse environment, as confirmed by RAPD analysis (not shown). This finding is difficult to explain because the leaves of the inoculated plants did not present symptoms and were not found in the leaf litter, where the fungi could release conidia. We observed an increase in the epiphytic fungal isolation frequency in the leaves of plants 20 days after inoculation with *E. nigrum*, but this effect did not persist as the plant aged (Figure 1c), which reflects the establishment of an equilibrium in the cultivable epiphytic fungal community or transient changes in the epiphytic fungal community as a result of the *E. nigrum* colonization. Moreover, an increase was also detected in the epiphytic fungal isolation frequency related to plant senescence (Figure 1c), which has been reported as a common phenomenon in studies on fungal diversity on plants [42].

**Figure 2:**
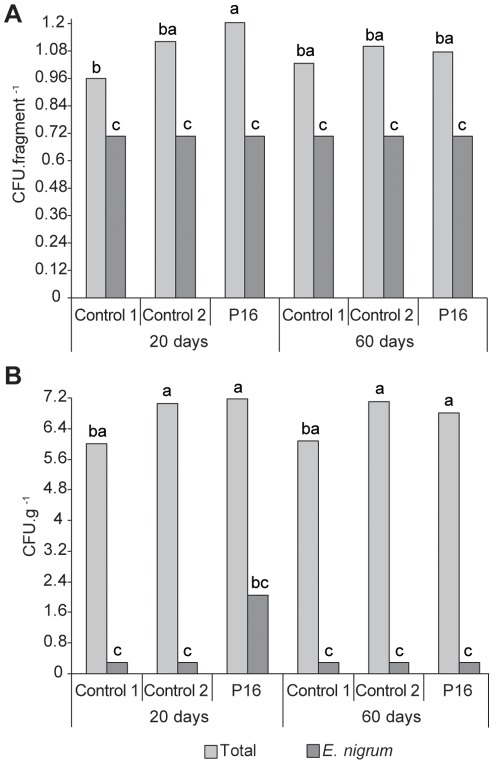
*E. nigrum* and sugarcane-associated fungi re-isolation from the root environment of sugarcane grown in greenhouse. *E. nigrum* and sugarcane-associated fungi were re-isolated 20 and 60 days after inoculation of the P16 endophytic strain in the roots of sugarcane plants. The *E. nigrum* isolation frequency was compared with the total isolation frequency of sugarcane-associated fungi. Control 1 indicates non-inoculated roots, while Control 2 indicates roots inoculated only with sterilized wheat seeds. Isolation frequency of the endophytic fungi from roots (a) is shown in CFU per root fragment. Isolation frequency of the rhizosphere fungi (b) is shown in CFU per gram of substrate. The data were transformed with √× + 0.5 (a) and Log (× + 2) (b) and submitted to analysis of variance followed by Tukey’s test. Means followed by the same letter indicate that they were not statistically different (Tukey’s test, *P>5%*).

*E. nigrum* was not recovered as an endophyte from inoculated sugarcane plants after root superficial disinfection (Figure 2a), which indicates that *E. nigrum* did not colonize the endophytic root environment under the analyzed conditions. However, 20 days after inoculation, we observed a significant increase in the total isolation frequency for fungi inside the sugarcane roots (Figure 2a), but this frequency declined 60 days after inoculation. *E. nigrum* was recovered from the rhizosphere only during the first isolation period (Figure 2b). These differences may have been caused by the establishment of a balance in the fungal population as the plants aged; however, the presence of *E. nigrum* may also have brought about transient alterations in the root endophytic fungal community, as observed for the leaf epiphytic environment. Importantly, the observed shift in the plant-associated fungal isolation frequency after *E. nigrum* inoculation may be the results of a combination of many factors, such as the type of inoculum and substrate [43], interspecific competition, the type of exudate released, and the chemical compounds on the leaf surface [10], [44].

Altogether, these results demonstrate that *E. nigrum* established a facultative endophytic interaction with sugarcane, preferentially in the phylloplane environment. This finding is in agreement with studies that have reported the isolation of this fungus mainly from sugarcane leaves [21], [22]. *E. nigrum* is considered to be a ubiquitous species in the epiphytic [28] and endophytic [29] environments and features characteristics such as melanized conidia, which increase UV tolerance and have been related to the capacity to colonize the phylloplane [37]. *E. nigrum* persistence in the phylloplane may be related to the different physiological conditions present in the plant tissues, but studies on other fungi have shown that there may be a preference for the tissue and conditions present in the studied plant organ [43]. These results demonstrate the importance of this approach for studying the interaction between endophytes and the microbial communities associated with the plant.

### *E. nigrum* Colonizes Sugarcane Leaves Through Natural Openings and Uses the Epidermis as a Preferential Niche

**Figure 3:**
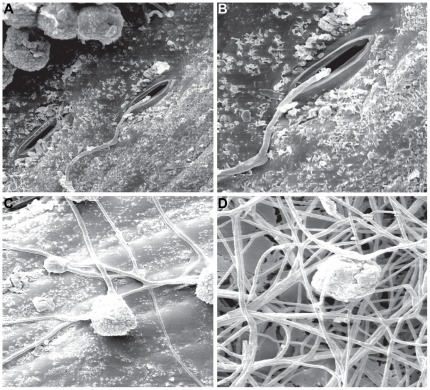
*E. nigrum* conidia germination on sugarcane leaf fragments analyzed by scanning electronic microscopy. Scanning electronic microscopy analysis of the conidia germination of the *E. nigrum* P16 endophytic strain on sugarcane leaf fragments. (a–b) After 12 hours of incubation in wet chamber it was possible to visualize the conidia germination and hyphae next to the stomata (1000X and 2000X, respectively). (c) After 40 hours of incubation it was possible to visualize hyphal ramification and random surface colonization (1000X). (d) After 64 hours, the leaf surface was completely covered with *E. nigrum* hyphae (1000X).

The germination of *E. nigrum* conidia on sugarcane leaf fragments was investigated by scanning electronic microscopy (SEM), which showed that conidia germination occurred at 12–16 hours after inoculation and that the hyphae penetrated the leaf tissue through the stomata (Figure 3). No changes associated with the direct penetration of the surface, such as the development of structures similar to the appressorium or changes on the leaf surface to which the conidia and hyphae were attached, were observed. Random hyphal ramification was observed 40 hours after surface colonization, and the hypha seems to firmly adhere to the cuticle (Figure 3). After 64 hours, the leaf surface was completely covered with *E. nigrum* hyphae (Figure 3).

The absence of direct penetration and changes on the leaf surface was also observed in the leaf disks of bean plants colonized by *E. nigrum* conidia [41], which suggests that this fungi is not able to induce disease symptoms in bean plants, as we showed in sugarcane. However, other studies are needed to determine whether the internal parenchyma is colonized or whether the fungus is restricted to the sugarcane epidermal intercellular spaces. In a recent assessment of the capacity of several *E. nigrum* endophytic isolates obtained from healthy *S. officinarum* leaves to secrete hydrolytic enzymes, high lipase production was observed, including for the P16 strain used in the present study [21]. Therefore, the colonization pattern observed by microscopy and the high lipase secretion suggest that the sugarcane epidermis is a preferential niche for this fungus, as was shown by the previous re-isolation analyses.

Among the endophytic fungi, there are many epiphyte species that belong to ubiquitous genera that can live inside the host plant [29]. It has been suggested that endophytic communities contain epiphyte species that show facultative leaf penetration, such as *Alternaria*, *Cladosporium* and *Epicoccum* [30]. However, studies have not been performed to test these hypotheses, and the analyses performed demonstrated that facultative endophytism is part of the *E. nigrum* life strategy. Furthermore, the occurrence of this fungus inside various, taxonomically unrelated plants [21], [30], [31], [32], [36], [45], [46], [47] suggests the development of adaptations to overcome different types of plant defenses, which is characteristic of a generalist lifestyle.

### *E. nigrum* Colonizes Sugarcane Asymptomatically and Increases Root System Biomass

Conidia of the *E. nigrum* P16 strain were inoculated in axenic sugarcane plants to investigate the possible effects of this fungus on plant survival in the *in vitro* rooting phase. At 72 hours of incubation, a mycelial film around the roots was observed (not shown). The general characteristics of the colonized plants were not altered compared with the control, and plant senescence was not postponed, which demonstrates the non-pathogenic character of the P16 strain.

Because *E. nigrum* P16 was not able to induce disease symptoms in *in vitro* propagated sugarcane plantlets, we investigated the effect of this fungus on the greenhouse acclimatization of sugarcane plants. This analysis was performed because the acclimatization process can be a stress period for plants [48] and is an opportune time to introduce protective microbial inoculants [49]. We observed no difference in the survival of inoculated plants after the acclimatization period compared with the control, which confirms that *E. nigrum* sugarcane colonization in this period is also asymptomatic. The absence of pathogenicity in *Epicoccum* endophytic isolates was demonstrated previously when strains of this fungus were re-introduced in *in vitro* propagated pejibaye plants to promote plantlet growth [46] and when pathogenicity tests were carried out in *Quercus* spp. plants for the use of *E. nigrum* in *Diplodia corticola* control [45]. More recently, an endophytic apple tree *E.* *nigrum* isolate was inoculated in *C. roseus* plants to control phytoplasma dissemination, and no disease symptoms were detected in pathogenicity tests with *C. roseus* plants inoculated with the *E. nigrum* isolate [36].

We further analyzed the effect of *E. nigrum* colonization of axenic sugarcane roots on plant growth after the acclimatization process by analyzing the accumulation of root and canopy fresh and dry matter. After 60 days of growth in a greenhouse, the dry matter accumulation in the roots of the colonized plants was greater than that in the control plants as demonstrated by the analysis of variance (Table 1), which suggests that this fungus increased the rooting capacity of the plant at this post-acclimatization phase. Although inoculation of the P16 strain tended to decrease the canopy biomass, significant differences were not found for the total dry matter of the inoculated plants compared with the control plants (Table 1). Root colonization by *E. nigrum* may have changed the carbon and dry matter distribution among the different parts of the plant because an increase in the root:canopy dry matter ratio was observed (Table 1), which indicates greater biomass allocation to the roots than to the canopy in the presence of *E. nigrum*. Importantly, before the acclimatization process, *E. nigrum* could not be recovered from the substrate (not shown), which indicates that the sugarcane root growth resulted from colonization by *E. nigrum*. However, we cannot discard other factors that could explain this effect, such as the alterations of the microbial community composition in the root environment, because in our previous experiments *E. nigrum* inoculation induced changes in the total isolation frequency of endophytic root fungi.

**Table 1:** Effect of the colonization of the *E. nigrum* P16 strain on the accumulation of root and canopy fresh and dry matter of the SP70-1143 sugarcane variety, after 60 days of growth in a greenhouse.

Treatment	Fresh weight (g)(1)				
	Roots	Aerialparts	Total	Root/aerial parts (2)	
SP70-1143 (control)	12.123^a^	14.118^a^	26.241^a^	0.86578^b^	
SP70-1143 (P16)	11.663^a^	10.664^b^	22.327^b^	1.09208^a^	
**Treatment**	**Dry weight (g)** (1)				
	**Roots**	**Aerial** **parts**	**Total**		**Root/** **aerial parts** (2)
SP70-1143 (control)	1.206^ba^	4.076^a^	5.282^a^		0.30269^b^
SP70-1143 (P16)	1.527^a^	3.039^b^	4.566^a^		0.50920^a^

(1) Data were submitted to analysis of variance followed by Tukey’s test. Means followed by the same letter indicate that they were not statistically different (Tukey’s test, *P>5%*, means from 10 replicates).

(2) Root:canopy dry matter ratio.

Although *E. nigrum* P16 induced a reduction of the canopy biomass, the increase in root systems could increase the adaptation of the inoculated plants in field conditions. Also, as disease symptoms were not observed *in vitro*, the presence of this fungus may increase plant fitness under specific conditions, such as in the presence of pathogens and/or pests, as has been reported for other interactions involving *E. nigrum* [36], [37], [50]. In fact, the results obtained in the present study indicate that physiological alterations could occur in the host plant as a result of *E. nigrum* colonization. Physiological and structural alterations, such as callose accumulation, have been observed in *C. roseus* plants inoculated with an endophytic apple tree *E. nigrum* strain to control phytoplasma symptoms [36], which indicates that physiological changes and bioprotective effects also could occur in non-host plants as a result of the *E. nigrum* colonization. Furthermore, the ability to produce plant growth-regulator-like molecules has been suggested to underlie the growth response to inoculation by endophytic fungi [1]. Plant hormone production of an *E. nigrum* endophytic strain has been observed in the culture medium [51]. Therefore, if these compounds are produced during the interaction with the host plant, they could be involved in root growth such as that observed in the present study.

Indeed, little is known about the costs and benefits of the association of endophytes with tropical plants [6], [10], [52], [53], despite the remarkably common occurrence of this interaction. Interestingly, endophytes have been demonstrated to induce an alert state in plants that is characterized by an increased capacity to express basic defense responses following biotic and abiotic challenges [13], [54]. In fact, even for the fairly well-studied association between *Clavicipitaceae* fungi and temperate climate grasses (*Poaceae*), some beneficial effects, such as the growth response of the plants to the presence of endophytes, are variable and depend on the host genotype, nutrient availability and environmental stresses [13]. Although further analyses are needed to assess the reproducibility of the effect of *E. nigrum* P16 on sugarcane root growth by addressing different conidia concentrations, sugarcane varieties, types of stress, and plot development strategies, colonization by endophytic fungi may increase the cost to the plant, as demonstrated by the physiological alterations resulting from asymptomatic *E. nigrum* colonization.

### Sugarcane Endophytic *E. nigrum* Inhibits Several Plant Pathogens

**Figure 4:**
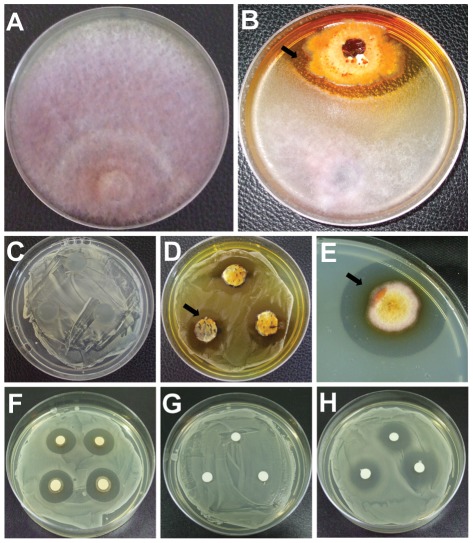
*In vitro* antagonism of the *E. nigrum* P16 endophytic strain against sugarcane phytopathogens. Antagonism test between *E. nigrum* P16 and *F. verticillioides* (a–b). An inhibition zone (dark arrow) formed among the colonies can be observed (b), in comparison with the control plate with only *F. verticillioides* (a). The inhibitory activity against *X. albilineans* was observed by the agar block method (c–d) and by the method of diffusion in semi-solid agar (e). The *E. nigrum* P16 ethyl acetate extract also inhibited *X. albilineans* growth (f–h). DMSO was used as control treatment (g). Spectinomycin (50 mg.mL^−1^) was used as positive control (h).

Endophytic fungi are known to protect plants against several biotic stresses, in part via production of secondary metabolites with biological activities. Therefore, we investigated the antagonistic potential of the sugarcane endophytic *E. nigrum* P16 strain against different phytopathogens. *E. nigrum* reduced *C. paradoxa* and *F.* *verticillioides* radial growth by more than 50% (Table 2), and an inhibition zone formed among the colonies that was stable even after 20 days of culture (Figure 4a–b), which indicates that diffusible compounds were released in the culture medium by the antagonistic endophyte. The inhibitory activity against *X.* *albilineans* was observed by the agar block method (Figure 4c–d), which yielded a 22.6 mm (±0.1 mm) inhibition zone, and by the method of diffusion in semi-solid agar (Figure 4e), which yielded a 9.0 mm (±0.1 mm) inhibition zone. These findings demonstrate that bioactive compounds were produced in the initial and the more advanced stages of *E. nigrum* growth on solid media.

**Table 2:** Antagonism test of the sugarcane endophytic *E. nigrum* P16 strain against the sugarcane phytopathogens *C. paradoxa* and *F. verticillioides.*

Treatment	*C. paradoxa*			*F. verticillioides*			
	Radialgrowth	Growthreduction	Inhibitionzone	Radialgrowth	Growthreduction	Inhibition zone	
Control	75 mm^a^	–	–	75 mm^a^	–	–	
P16	31 mm^b^	58.6%	4.5 (±0.1 mm)	34.5 mm^b^	54%	4 (±0.1 mm)	

Data were submitted to analysis of variance followed by Tukey’s test. Means followed by the same letter indicate that they were not statistically different (Tukey’s test, *P>5%*, means from 3 replicates).

**Figure 5:**
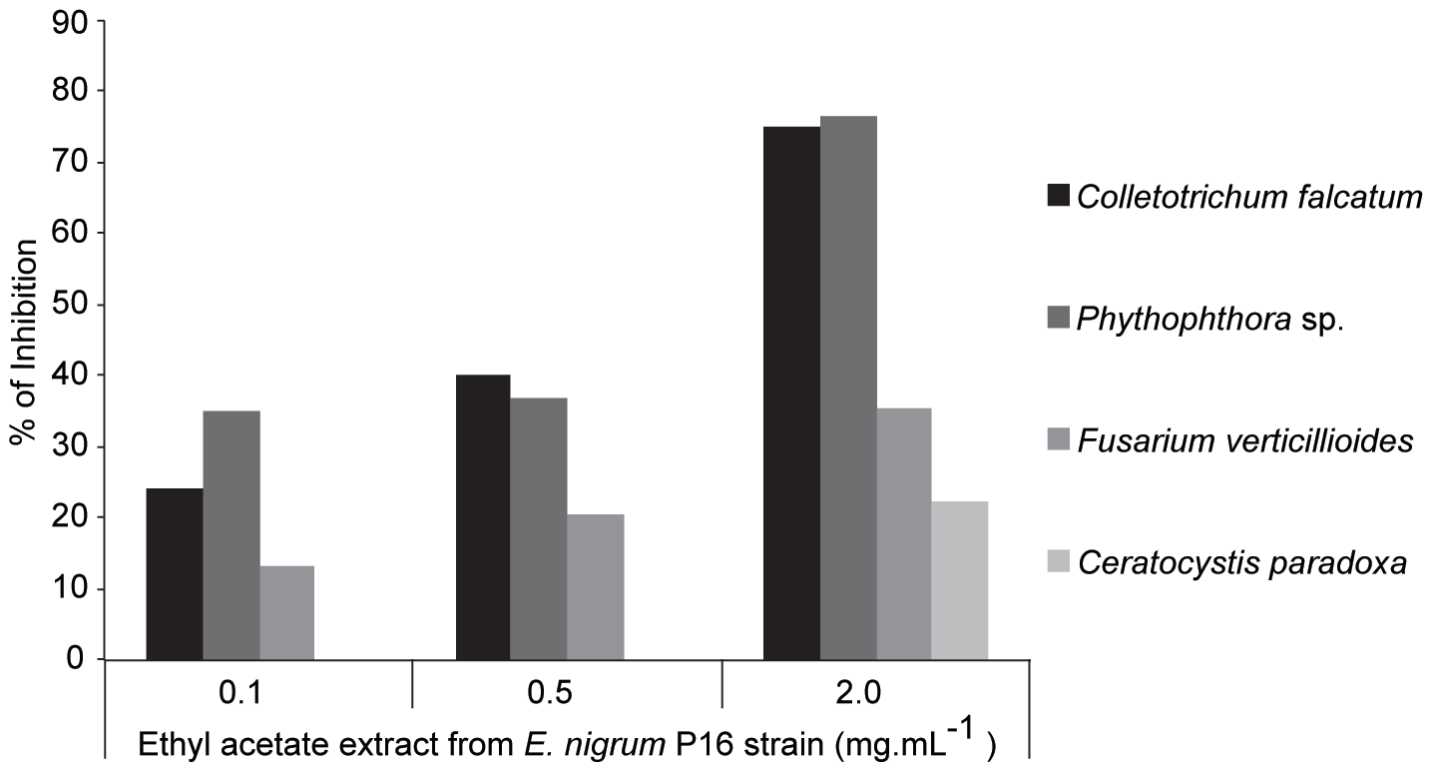
Antimicrobial activity of the ethyl acetate extract from *E. nigrum* P16 endophytic strain. Antifungal and anti-oomycete activity of the ethyl acetate extract from *E. nigrum* P16 strain. The percentage of growth inhibition of the pathogens is showed in the y axis. The means of three replicates for each extract concentration analysed were used to calculate the percent reduction in pathogen mycelial growth by the equation [1– (mean colony diameter of the control/mean colony diameter of the treatment)×100].

Because we observed that diffusible compounds were produced during *E. nigrum* growth in solid medium, we also investigated if the organic extracts of the supernatant (ethyl acetate) and the mycelium (methanol) of the *E. nigrum* culture had inhibitory activity. The extract obtained from the mycelium did not exhibit antimicrobial activity under the assessment conditions, possibly because the compounds were not stored in the mycelia or their production was low and their presence could not be detected by the methods used. However, the ethyl acetate extract inhibited *X.* *albilineans* growth (Figure 4f–h) and produced a 15.5 mm (±0.1 mm) inhibition zone; the inhibition zone produced by the antibiotic spectinomycin, which was used as a positive control, was 19.0 mm (±0.1 mm). The *E. nigrum* extract also significantly reduced *C.* *falcatum*, *F. verticillioides*, *C. paradoxa* and *Phythophthora* sp. growth at concentrations ranging from 0.1 to 2.0 mg.mL^−1^. *C. falcatum* and *Phythophthora* sp. were more sensitive to the extract, with colony diameter reductions of 75% and 76.47%, respectively, in the presence of 2.0 mg.mL^−1^ of the extract (Figure 5).

The antagonistic activity of *E. nigrum* has already been demonstrated against different fungal and oomycete phytopathogens [34], [35], [39], [45], [47], [55], [56], [57], [58], [59], [60], [61], [62], [63]. Some antimicrobial compounds produced by *E. nigrum* have been characterized, such as epicorazines A–B [64], epirodines A–B [65], flavipin [66], epicoccines A–D [67], epipiridones and epicocarines [68]. In particular, flavipin and epicorazines A–B have been associated with *E. nigrum* biocontrol activity against *Monilinia* spp., *Pythium* ssp. and *Phythophthora* ssp. [55], [69], [70]. In addition, flavipin also inhibits plant pathogenic bacteria such as *Corynebacterium michiganense*, *Erwinia carotovora* var. *atroseptica*, *Pseudomonas phaseolicola*, *P. putida*, *P. syringae* and *X. phaseoli* [69], which illustrates the broad spectrum of action of this metabolite. Therefore, the common sugarcane endophyte *E. nigrum* [21], [22], [23] may act as a natural antagonist for several sugarcane pathogens if it produces these compounds during the interaction with the host plant.

### Concluding Remarks

A study of the basic aspects of the interaction of *E. nigrum* with sugarcane demonstrated the facultative endophytism of *E. nigrum* and its preference for the phylloplane environment, which should be considered in future studies of biocontrol using this species. Furthermore, an increase in the root system biomass was observed in plants inoculated with *E. nigrum*, which demonstrates the need for greater investigation of the physiological alterations and molecular mechanisms involved in the symbiosis. Endophytic fungi have received increasing interest as a promising source of potential control agents against plant pathogens [10], [11]. Although endophytes have potential uses in agriculture, the incomplete understanding of the biology of the endophyte-plant interaction impedes their widespread use [2], [13]. For example, with the exception of the *Clavicipitaceae* endophytes, little is known about the function of the secondary metabolites in the endophyte-plant interaction. The present study demonstrates that the sugarcane leaf endophyte *E. nigrum* inhibited the *in vitro* growth of different microorganisms, which indicates that this endophyte could be a natural antagonist for plant pathogens in sugarcane tissues. These findings suggest that the plant interior is a prolific environment for discovering *E. nigrum* strains that may produce new metabolites and opens the possibility of characterizing different isolates of this fungus to select more promising strains. In this context, a new natural product, epicolactone, was recently isolated from the ethyl acetate extract of the *E. nigrum* P16 strain analyzed in the present work [71].

The compounds produced by *E. nigrum* that are responsible for inhibiting plant pathogens must be characterized. Although *E.* *nigrum* has a fairly diverse secondary metabolism, there are no genetic studies on the biosynthesis of bioactive compounds. Our results should encourage future studies to evaluate secondary metabolite synthesis by *E. nigrum* and define the best conditions for using this fungus in sugarcane culture. Such studies are underway in our laboratory and have been facilitated by the optimization of genetic transformation protocols [24] and the complete genomic sequencing of the *E. nigrum* P16 strain, which was recently approved by our group (FAPESP Grant 10/08286-2 - BioProject Accession PRJNA7784).

## Materials and Methods

### Strains, Growth Conditions, and Conidia Production

The *E. nigrum* P16 strain was isolated from surface-disinfected healthy sugarcane leaves [21] and maintained in Potato Dextrose Agar (PDA) (Difco). To obtain conidia, *E. nigrum* was inoculated on sterile sugarcane leaf fragments on Petri dishes containing agar-agar (1.5% p/v). After incubation for 25 days at 28°C with a 16-hour light period, a conidia suspension was prepared (1×10^6^ conidia.mL^−1^). *Fusarium verticillioides*, *Ceratocystis paradoxa*, *Colletotrichum falcatum* (sugarcane pathogens), and *Phythophthora* sp. (*Citrus* sp. pathogen) were maintained in PDA. *Xanthomonas albilineans* (sugarcane pathogen) was maintained in Nutrient Agar (NA) (Difco). The pathogens were obtained from the collection of microbial strains in the Laboratory of Microbial Genetics, Department of Genetics, ESALQ/USP, Piracicaba, São Paulo, Brazil.

### *E. nigrum* Inoculation in Sugarcane Seedlings

Thirty-day-old sugarcane plants (SP80-1842, conventional variety) were grown on trays with PlantMax commercial substrate (Eucatex, Brazil) and kindly supplied by the Sugarcane Technology Center (Centro de Tecnologia Canavieira S.A.). To inoculate the roots, 20 g wheat seed was placed in flasks, moistened with 10 mL distilled water and sterilized in an autoclave three consecutive times. Two *E. nigrum* mycelia disks were then inoculated over the seeds and incubated at 28°C for 15 days. The 1-kg pots were filled with PlantMax commercial plant substrate, and the seedlings were transferred to these pots with 20 g of the previously prepared inoculum so that the seeds colonized by the fungus were in contact with the roots. A randomized block design was used with the three treatments (SP80-1842 - not inoculated, SP80-1842+ wheat seeds, SP80-1842+ *E. nigrum* P16 strain) with three replicates. This experimental design was considered for the analysis of variance (ANOVA). The inoculum was prepared by transferring three mycelia disks to flasks containing 200 mL potato broth culture medium (12 flasks). After growth for 15 days at 28°C, the mycelia were filtered, and the mass was estimated. The mycelia were then mixed with PBS buffer (8 g NaCl, 0.2 g KCl, 1.4 g Na_2_HPO_4_, 0.24 g KH_2_PO_4_, 1.000 mL distilled water, pH 7.4) at a proportion of 70 g.L^−1^, homogenized and inoculated on the sugarcane leaves. The plants were kept under highly humid conditions in a greenhouse for 48 hours (wet chamber made with transparent plastic bags). Application of the PBS buffer was used as the control. A randomized block design was used with two treatments (SP80-1842 not inoculated, SP80-1842+ *E. nigrum* P16 strain) and three replicates, in which each plant was considered a replicate. This experimental design was considered for the analysis of variance (ANOVA).

### Re-isolation of *E. nigrum* from Sugarcane Plants

*E. nigrum* and sugarcane-associated fungi were re-isolated 20 and 60 days after inoculation. For the re-isolation, the endophytic fungal community from the leaves, sheath, and roots and the phylloplane epiphytic and rhizosphere fungal communities were isolated. The leaf and sheath endophytes were isolated after superficial disinfection (70% ethanol for 60 seconds, 3% sodium hypochlorite (v/v) for 90 seconds, 70% ethanol for 60 seconds and rinsed twice with sterilized water). Seven leaf fragments (0.5 cm^2^) were transferred to Petri dishes containing PDA supplemented with tetracycline (50 µg mL^−1^).

For the isolation of root endophytes, the roots were washed in running water, and a 2-g sample was disinfected superficially (70% ethanol for 60 seconds, 3% sodium hypochlorite (v/v) for 180 seconds, 70% ethanol for 60 seconds and rinsed twice with sterilized water). To assess the disinfection efficiency, a 100-µL aliquot of the water used in the last wash was sown on the PDA culture medium. The plates were incubated at 28°C for 5–15 days, and the number of colonies was converted to colony forming units (CFUs) per fragment. The data were transformed with √×+0.5 and submitted to analysis of variance and the Tukey test at the level of 5% significance using SAS software (Copyright (c) 1989–1996 by SAS Institute Inc., Cary, NC, USA).

To isolate the fungi from the phylloplane surfaces, ten leaf fragments (5.0×1.5 cm^2^) were transferred to flasks containing glass spheres (0.2 cm diameter) and 50 mL PBS buffer. After shaking for 2 hours (200 rpm) at 28°C, 100 µL aliquots were sown on PDA supplemented with tetracycline (50 µg mL^−1^) and incubated at 28°C for 7 days. The number of fungal colonies was converted to CFUs per square centimeter based on the upper and lower surfaces of the leaf fragments used. These data were transformed with √×+0.5 and submitted to analysis of variance and the Tukey test at 5% significance using SAS software (Copyright (c) 1989–1996 by SAS Institute Inc., Cary, NC, USA).

To isolate the fungi from the rhizosphere, the excess extract was removed, the roots were shaken vigorously to collect the substrate adhering to them, and 5-g samples were transferred to Erlenmeyer flasks containing glass spheres and 50 mL PBS buffer. After incubation for one hour at 28°C under agitation (200 rpm), the dilutions were sown on PDA supplemented with tetracycline (50 µg mL^−1^) and incubated at 28°C for 5 days. The number of CFUs per gram of extract was transformed with log (×+2) and submitted to analysis of variance and the Tukey test at 5% significance using SAS software (Copyright (c) 1989–1996 by SAS Institute Inc., Cary, NC, USA).

### Genetic Identity of *E. nigrum* Re-isolates

The colonies obtained in the isolation that were morphologically similar to *E. nigrum* were compared with the original P16 strain using RAPD markers [72]. For the comparison, three mycelia disks from monoconidial colonies of the sugarcane-recuperated isolates were transferred to flasks containing 50 mL potato broth. After 7 days of growth at 28°C, the mycelia were collected by filtration, and the genomic DNA was extracted with the Wizard Genomic DNA Purification Kit (Promega, USA) and used as a template in PCR reactions.

The OP-X12 (5′ TCGCCAGCCA 3′), OP-X17 (5′ GACACGGACC 3′) and OP-X19 (5′ TGGCAAGGCA 3′) oligonucleotides (Operon Technologies, USA) were used in the RAPD reactions and prepared in duplicate to a final volume of 25 µL with 0.25 mM dNTPs, 3.0 mM MgCl_2_, 1× buffer (50 mM KCl and 20 mM Tris-HCl, pH 8.4), 0.5 U.µL^−1^ Taq DNA polymerase (Fermentas Life Sciences, Brazil), 0.4 µM each primer/starter and 5 ng genomic DNA. The amplification was performed in a PTC - 200 thermocycler (MJ Research) with an initial denaturation at 94°C for four minutes followed by 40 amplification cycles. Each cycle consisted of one minute at 92°C, one minute at 35°C, two minutes at 72°C and a final extension of five minutes at 72°C. The PCR products were separated in a 1.5% agarose gel, stained with ethidium bromide and photodocumented under ultraviolet light.

### Colonization of Sugarcane Leaf Fragments

To investigate the conidia germination of the P16 strain on sugarcane leaf fragments, four–month-old plant leaves of the SP80-1842 variety grown at the Sugarcane Technology Center (Centro de Tecnologia Canavieira S.A.) were superficially disinfected (70% ethanol for 60 seconds, 3% sodium hypochlorite (v/v) for 90 seconds, 70% ethanol for 60 seconds and rinsed twice with sterilized water) and transferred to a wet chamber (Petri dishes containing filter paper moistened with sterilized distilled water). Aliquots of 10 µL of a conidia suspension (1×10^5^ conidia.mL^−1^) were inoculated on the abaxial surface of the leaf fragments. The plates were incubated at 28°C with a 16-hour photoperiod, and 5-mm leaf fragments were collected at regular intervals (0 h, 6 h, 12 h, 18 h, 24 h, 30 h, 36 h, 42 h, 48 h and 72 h) and fixed in Karnovsky solution (2.5% glutaraldehyde, 2.5% formaldehyde in 0.05 M sodium cacodylate, pH 7.2, 0.001 M CaCl_2_). The samples were fixed with osmium tetroxide (1% OsO_4_ in 0.1 M cacodylate buffer), dehydrated in ethanol solutions with increasing concentrations (30, 50, 70, 90 and 100%) and then dried to the critical point and metallized. The analysis was performed with a Zeiss DSM 940 A scanning electronic microscope at the Research Support Nucleus for Electronic Microscopy (NAP/MEPA, ESALQ/USP, Piracicaba, SP).

### Greenhouse Evaluation of *E. nigrum* on Sugarcane Growth

Axenic plants of the SP70-1143 variety at the rooting stage were donated by the Sugarcane Technology Center (Centro de Tecnologia Canavieira S.A.). First, the general aspect of the plants was assessed visually. The conidia of the P16 strain were inoculated in flasks containing 20 mL Murashige and Skoog (MS) culture medium [73], and the concentration was adjusted to 1×10^5^ conidia.mL^−1^. The plants were transferred to the flasks, and the roots were immersed in the culture medium and incubated at 28° with a 16-hour photoperiod. The plants were inspected visually at 24-, 48-, 72- and 96-hour intervals (three plants for each interval) or until the non-inoculated plants entered a state of senescence.

Subsequently, 50-mL Falcon type tubes were prepared containing 7 mL of MS culture medium [73] inoculated with 1×10^5^ conidia.mL^−1^. Micropropagated plantlets with homogeneous characteristics (same canopy size and similar root number in the *in vitro* culture) were individualized and aseptically transferred to the tubes containing conidia. The plants were incubated at 28°C with a 16-hour photoperiod for three days and were then acclimatized in a greenhouse. For acclimatization, plastic trays with 200-mL wells were filled with commercial PlantMax substrate. The plantlets were transplanted to the substrate and maintained in a wet chamber under greenhouse conditions for 10 days.

After this period, the wet chamber was removed, and the plants were watered every two days with 50 mL water. After a 60-day growth period under greenhouse conditions, the plants were collected and assessed for fresh and dry matter root and canopy accumulation. *E. nigrum* was also re-isolated and analyzed by RAPD, as reported previously. To measure the dry matter, the plants were incubated in a chamber at 80°C for 24 hours and then at 65°C until a constant weight was reached. The analysis of variance was carried out with 10 plants from each treatment (SP70-1143 not inoculated; SP70-1143+ *E. nigrum* P16 strain) in a complete randomized design using SAS software (Copyright (c) 1989–1996 by SAS Institute Inc., Cary, NC, USA); the Tukey test was applied at the level of 5% significance.

### Antagonism Against Plant Pathogenic Microorganisms

*E. nigrum* and the plant pathogens were cultivated for 7 days at 28°C in PDA. *E. nigrum* mycelia disks (6 mm in diameter) were transferred to PDA medium 48 hours before inoculation with pathogens. The pathogens were inoculated 5 cm away from the *E.* *nigrum* P16 colony. As a control, the pathogen was inoculated without the *Epicoccum* colonies. After five days of incubation at 28°C, the inhibition zone and the percentage of growth inhibition of the pathogen were calculated in relation to the control. The tests were performed in triplicate.

*X. albilineans* inhibition was evaluated by the agar block method [74]. *E. nigrum* was cultured for 15 days in 15 mL PDA at 28°C. The bacterial culture was prepared in 10 mL nutrient broth (3 g meat extract, 5 g peptone, 1000 mL distilled water, pH 6.8) and incubated with agitation (100 rpm) for 24 hours at 30°C. An aliquot (50 µL) of this culture was sown on 15 mL NA (Difco), and *E. nigrum* disks (8 mm diameter) were then transferred to the dishes containing the bacteria. The inhibition halo was measured after 24 hours of growth at 30°C. The control consisted of inoculating the PDA disks, and the bioassay was performed in triplicate.

Activity against *X. albilineans* was also investigated using a previously reported method [75]. *E. nigrum* fragments were inoculated in 10 mL PDA, and the dishes were incubated for 72 hours at 28°C in the dark. *X. albilineans* was cultured as described previously, and one aliquot of the culture (1%, v/v) was transferred to semisolid NA (0.5% agar; 45°C–50°C). Then, 7 mL of this culture medium was poured over the *E. nigrum* cultures, and the dishes were stored for 8 hours at 4°C before incubation at 30°C for 24 hours. The tests were performed in triplicate, and the inhibition zone was then measured.

### Antimicrobial Activity of the Organic Extract of *E.* *nigrum* Cultures

To produce the bioactive compounds in liquid culture medium, 3 mycelia disks (8-mm diameter) from *E. nigrum* cultures grown on PDA for 7 days at 28°C were inoculated in flasks containing 200 mL potato broth supplemented with 2% yeast extract (200 g boiled potato, 20 g glucose, 20 g yeast extract, 1000 mL distilled water, pH 6.8). The flasks were incubated at 28°C without light in static culture for 45 days. The mycelia and the fermentative culture medium were separated by filtering under vacuum, and the filtrate was submitted to three consecutive extraction steps with ethyl acetate. Ethyl acetate and the filtrate were mixed at a ratio of 1∶3 (v/v), and after agitation, the aqueous and organic phases were separated in a separation funnel.

The mycelia were immersed in flasks containing 100 mL dichloromethane and left at room temperature for 48 hours. The organic solvent was collected by filtration, and the mycelia were again immersed in 100 mL dichloromethane and methanol (1∶1), left to rest for 48 hours and then immersed in methanol alone for an additional 48 hours. The solvents were concentrated in a rotating evaporator under reduced pressure.

Aliquots of 20 µL of the organic extracts (100 mg.mL^−1^ in dimethylsulfoxide) were inoculated on 6-mm-diameter filter paper disks placed on the surface of Petri dishes containing culture medium inoculated with *X. albilineans,* as described previously. The experiment was performed in triplicate, and the control consisted of the inoculation of 20 µL dimethylsulfoxide and/or spectinomycin (Sigma-Aldrich) (50 mg.mL^−1^). The dishes were incubated at 30°C for 24 hours. The inhibition halo diameters were measured perpendicularly with a ruler. The ethyl acetate extract was also assessed against *F. verticillioides*, *C. paradoxa* and *C. falcatum*; the mycelial fragments of the pathogen were inoculated on Petri dishes (60×15 mm) containing 5 mL PDA, and increasing concentrations of the extract (0.1, 0.5, 2.0 mg.mL^−1^), and dimethylsulfoxide (control) alone were added. The colony diameters were measured after 4 days of incubation at 28°C. The experiment was performed in triplicate in a complete randomized design. The analysis of variance followed by Tukey’s test was conducted using SAS software (Copyright (c) 1989–1996 by SAS Institute Inc., Cary, NC, USA). The Tukey’s test was applied at the level of 5% significance. The percent reduction in pathogen mycelial growth was also calculated using the following equation: [1– (mean colony diameter of the control/mean colony diameter of the treatment) x 100].

## Supporting Information

Figure S1 RAPD profile generated with primer OPX12 (a), OPX17 (b), and OPX19 (c) of the original *E. nigrum* P16 strain (left) and six endophytic re-isolates obtained from sugarcane leaves variety SP80-1842, 20 days after inoculation in greenhouse. Amplification products were separated in 1.4% agarose gels and stained with ethidium bromide. (M) DNA ladder 1 Kb (Fermentas Life Sciences, Brazil).
(DOC)
