# Supplementary material for: Epicoccum nigrum P16, a Sugarcane Endophyte, Produces Antifungal Compounds and Induces Root Growth
Source: PLoS One. 2012 Jun 4;7(6):e36826. doi: 10.1371/journal.pone.0036826 (PMC3366970; doi:10.1371/journal.pone.0036826)
Supplement: Figure S1 — RAPD profile generated with primer OPX12 (a), OPX17 (b), and OPX19 (c) of the original E. nigrum P16 strain (left) and six endophytic re-isolates obtained from sugarcane leaves variety SP80-1842, 20 days after inoculation in greenhouse. Amplification products were separated in 1.4% agarose gels and stained with ethidium bromide. (M) DNA ladder 1 Kb (Fermentas Life Sciences, Brazil). (DOC) [file pone.0036826.s001.doc]

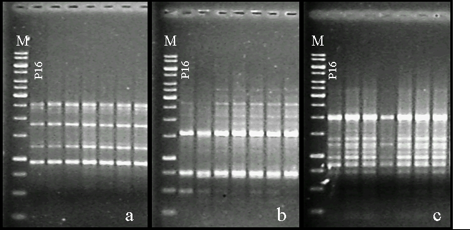


**Figure S1.** RAPD profile generated with primer OPX12 (a), OPX17 (b), and OPX19 (c) of the original *E. nigrum* P16 strain (left) and six endophytic re-isolates obtained from sugarcane leaves variety SP80-1842, 20 days after inoculation in greenhouse. Amplification products were separated in 1.4 % agarose gels and stained with ethidium bromide. (M) DNA ladder 1Kb (Fermentas Life Sciences, Brazil).
